# Interplay effect of angular dependence and calibration field size of MapCHECK 2 on RapidArc quality assurance

**DOI:** 10.1120/jacmp.v15i3.4638

**Published:** 2014-05-08

**Authors:** Hosang Jin, Vance P. Keeling, Daniel A. Johnson, Salahuddin Ahmad

**Affiliations:** ^1^ Department of Radiation Oncology University of Oklahoma Health Sciences Center Oklahoma City OK USA

**Keywords:** MapCHECK 2, angular dependence, RapidArc QA, calibration field size, VMAT QA

## Abstract

The purpose of this study is to investigate an effect of angular dependence and calibration field size of MapCHECK 2 on RapidArc QA for 6, 8, 10, and 15 MV The angular dependence was investigated by comparing MapCHECK 2 measurements in MapPHAN‐MC2 to the corresponding Eclipse calculations every 10° using 10 × 10 cm^2^ and 3 × 3 cm^2^ fields. Fourteen patients were selected to make RapidArc plans using the four energies, and verification plans were delivered to two phantom setups: MapCHECK 2/MapPHAN phantom (MapPHAN QA) and MapCHECK 2 on an isocentric mounting fixture (IMF QA). Migration of MapCHECK 2 on IMF was simulated by splitting arcs every 10° and displacing an isocenter of each partial arc in the Eclipse system (IMF_ACTUAL_ QA). To investigate the effect of calibration field size, MapCHECK 2 was calibrated by two field sizes (10 × 10 cm^2^ and 3 × 3 cm^2^) and applied to all QA measurements. The γ test was implemented using criteria of 1%/1 mm, 2%/2 mm, and 3%/3 mm. A mean dose of all compared points for each plan was compared with respect to a mean effective field size of the RapidArc plan. The angular dependence was considerably high at gantry angles of 90° ± 10° and 270° ± 10° (for 10 × 10/3 × 3 cm^2^ at 90°, 30.6% ± 6.6%/33.4%± 5.8% (6 MV), 17.3% ± 5.3%/15.0% ± 6.8% (8 MV), 8.9% ± 2.9%/7.8% ± 3.2% (10 MV), and 2.2% ± 2.3%/‐1.3% ± 2.6% (15 MV)). For 6 MV, the angular dependence significantly deteriorated the γ passing rate for plans of large field size in MapPHAN QA (< 90% using 3%/3 mm); however, these plans passed the γ test in ${\text{IMF}}_{\text{ACTUAL}}$ QA (> 95%). The different calibration field sizes did not make any significant dose difference for both MapPHAN QA and ${\text{IMF}}_{\text{ACTUAL}}$ QA. For 8, 10, and 15 MV, the angular dependence does not make any clinically meaningful impact on MapPHAN QA. Both MapPHAN QA and ${\text{IMF}}_{\text{ACTUAL}}$ QA presented clinically acceptable γ passing rates using 3%/3 mm. MapPHAN QA showed better passing rates than ${\text{IMF}}_{\text{ACTUAL}}$ QA for the tighter criteria. The 10 × 10 cm^2^ calibration showed better agreement for plans of small effective field size (< 5 × 5 cm^2^) in MapPHAN QA. There was no statistical difference between IMF QA and ${\text{IMF}}_{\text{ACTUAL}}$ QA. In conclusion, MapPHAN QA is not recommended for plans of large field size, especially for 6 MV, and MapCHECK 2 should be calibrated using a field size similar to a mean effective field size of a RapidArc plan for better agreement for IMF QA.

PACS numbers: 87.55.km, 87.55.Qr, 87.56.Fc

## I. INTRODUCTION

Volumetric‐modulated arc therapy (VMAT) has rapidly become routine radiation therapy due to improved efficiency of delivery and reduced treatment time with comparable dose distribution to conventional intensity‐modulated radiation treatment (IMRT).^1^, ^2^, ^3^, ^4^, ^5^, ^6^ RapidArc (Varian Medical Systems, Palo Alto, CA) has become available for the arc‐dynamic VMAT, which consists of continuous gantry rotation and variations of dose rate and gantry speed while radiation fields shaped by multileaf collimator (MLC) are dynamically changing. This intrinsic complexity of RapidArc makes pretreatment quality assurance (QA) onerous. Especially, subfields of RapidArc are composed of multiple small isolated apertures (less than $1\times 1\;{\text{cm}}^{2}$) within each control point which requires small‐scale (submillimeter) detectors in a high‐density, two‐dimensional (2D) or three‐dimensional (3D) array.

Common practice of VMAT QA depends on conventional IMRT QA devices. Chandraraj et al.^(7)^ compared the dosimetric performance of Kodak EDR2 film and three QA devices (Im'RT MatriXX array, IBA Dosimetry, Schwrazenbruck, Germany; PTW seven29 array, PTW‐Freiburg, Germany; and Scandios Delta^4^ array, ScandiDos, Uppsala, Sweden) for the VMAT QA. All patient QAs (15 RapidArc plans) passed the tolerance of 90% of points having gamma index less than 1.0 with the four QA systems. Zhu et al.^(8)^ also compared four different QA devices for the VMAT QA — electronic portal imaging device (EPID), Seven29, Im'RT MatriXX, and Delta^4^ — and presented similar results. Li et al.^(9)^ tested ArcCHECK from Sun Nuclear Corporation (SNC, Melbourne, FL) for IMRT and VMAT QAs and showed that the ArcCHECK QA system is suitable for clinical IMRT and VMAT verification. Multiple research groups demonstrated that the EPID portal dosimetry is also a valid QA tool for the verification of VMAT fields.^10^, ^11^, ^12^ Sun Nuclear's MapCHECK or MapCHECK 2 is one of the most widely used IMRT QA devices. Application of these devices for VMAT QA has drawn many physicists' attention due to diode size (0.8mm×0.8mm) ideal for small fields, linear response, reproducibility, convenience of setup, and real‐time analysis.^13^, ^14^, ^15^ Gloi et al.^(16)^ showed that the MapCHECK system is a useful QA tool for VMAT (RapidArc) therapies of partial arcs. Rinaldin and colleagues^(17)^ achieved the mean percentage with γ index >95% equal to 90.3% for 386 RapidArc patients, excluding a high contribution of lateral fluence using MapCHECK 2. Jursinic et al.^(18)^ concluded that MapCHECK can be used for patient‐specific QA measurements delivered with rotational IMRT techniques, such as helical TomoTherapy and VMAT (RapidArc), if the MapCHECK response becomes isotropic with proper modification.

Performance of diode array‐based detectors, such as MapCHECK/MapCHECK 2 and Scandios Delta^4^, is limited by intrinsic dosimetric drawbacks of diode such as energy dependence, differential response to scattered radiation, and angular dependence of dose sensitivity.^(19)^ Several research groups^18^, ^20^ showed about 25% of overresponse of MapCHECK diode for 6 MV with the beam incidence angles of $90^{\circ }(270^{\circ })\pm 5^{\circ }$. In addition, MapCHECK 2 (calibration using the standard $10\times 10\;{\text{cm}}^{2}$ field) underestimates dose when the field sizes are small due to higher sensitivity of diode to scattered radiation (∼1% for $2\times 2\;{\text{cm}}^{2}$ field using 6 MV beam).^(21)^ Even if a number of research efforts have been made to remedy the angular sensitivity,^18^, ^22^, ^23^ few research provides a complex interplay of the angular dependence and the effect of calibration field size using different beam energies for RapidArc plan verification with MapCHECK 2. The goal of this study is to investigate the effect of the angular dependence and different calibration field sizes of MapCHECK 2 on the RapidArc QA using four beam energies.

## II. MATERIALS AND METHODS

### A. Angular dependence of MapCHECK 2 diode

**Figure 1:**
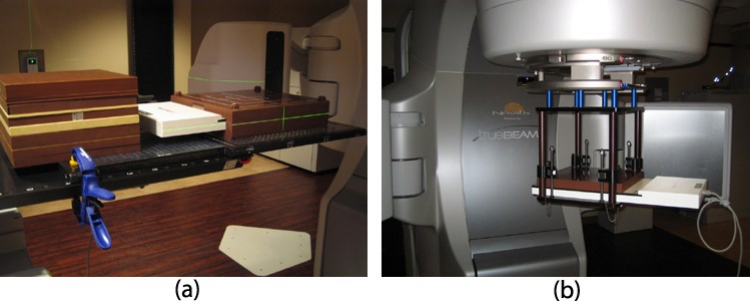
MapCHECK 2 device set up for measuring IMRT validation plans: (a) MapPHAN QA using MapPHAN‐MC2 on an extended tennis‐racket‐type grid couch top, and (b) IMF QA using the isocentric mounting fixture (IMF).

The angular dependence of MapCHECK 2 was investigated by comparing measurements on a tennis‐racket‐type grid couch top to calculations by the Varian Eclipse treatment planning system (TPS; version 11.0) as shown in Fig. 1(a). All radiation beams were delivered by the Varian TrueBeam STx that was commissioned for four beam energies (6, 8, 10, and 15 MV) and equipped with high definition MLC. The linac couch was not modeled in the TPS to eliminate any dosimetric uncertainty from the couch modeling. The MapCHECK 2 with MapPHAN‐MC2 (water‐equivalent buildup of 5.0 cm above and below the detector plane) was scanned (512×512 matrix with pixel size of 0.78 mm; slice thickness of 2.5 mm) using a GE CT scanner (GE Medical Systems, San Francisco, CA) and transferred to the Eclipse TPS for dose calculation. The dose was computed using anisotropic analytical algorithm (AAA) with a calculation grid of 2 mm. The raw CT scan without any postprocessing or correction for asymmetric diode sensitivity was used for the treatment planning to simulate a worst‐case scenario. The measurements were performed for every 10° gantry angle using four energies and two field sizes ($3\times 3\;{\text{cm}}^{2}$ and $10\times 10\;{\text{cm}}^{2}$). MapCHECK 2 was calibrated by a standard protocol using a $10\times 10\;{\text{cm}}^{2}$ field instructed by the vendor. A mean percent dose difference ((Measurement−Calculation)/(Calculation×100%)) and a standard deviation (SD) of comparison points (five diode points within $1\times 1\;{\text{cm}}^{2}$ around the isocenter for $3\times 3\;{\text{cm}}^{2}$ field, and 13 points within $2\times 2\;{\text{cm}}^{2}$ for $10\times 10\;{\text{cm}}^{2}$ field) were calculated for each measurement. The comparison was implemented in an in‐house computer program coded by MATLAB (MathWorks, Inc., Natick, MA).

### B. RapidArc verification plans and delivery

RapidArc treatment plans were generated by the Eclipse TPS for 14 patients selected from different treatment sites, as summarized in Table 1. The radiotherapy plans were made using four energies with the same optimization objectives for each patient. The patient‐specific QA plans were generated without changing any beam parameters on the MapCHECK 2/MapPHAN scan. These plans are substantially affected by the angular dependence of MapCHECK 2 diode during QA delivery. The verification plans were delivered to the MapCHECK 2/MapPHAN‐MC2 system (calibrated using the $10\times 10\;{\text{cm}}^{2}$ field) on the extended grid couch (referred as “MapPHAN QA” in this study).

**Table 1:** Summary of the RapidArc QA patients

*Patient Number*	*Treatment Site*	*No. of Arcs*	*Angle of Arcs (direction)*	*Gantry Start*	*Gantry Stop*	*Collimator Angle*
1	Brain	2	120°(CW), 90°(CW)	200°, 230°	320°, 320°	45°, 315°
2	Brain	1	358.9° (CW)	180.1°	179°	0°
3	Chest	1	210° (CW)	300°	150°	^45°^
4	Head and Neck	1	358.9° (CW)	180.1°	179°	45°
5	Head and Neck		358.9° (CW), 358.9° (CW)	180.1°, 180.1°	179°, 179°	330°, 30°
6	Head and Neck	1	358.9° (CW)	180.1°	179°	45°
7	Lung	1	358.9° (CW)	180.1°	179°	45°
8	Lung		358.9° (CW), 358.9° (CCW)	180.1°, 179°	179°, 180.1°	45°, 315°
9	Pancreas	1	358.9° (CW)	180.1°	179°	45°
10	Prostate	1	358.9° (CW)	180.1°	179°	45°
11	Prostate	1	358.9° (CW)	180.1°	179°	45°
12	Rectum	1	358.9° (CW)	180.1°	179°	45°
13	Stomach	1	358.9° (CW)	180.1°	179°	45°
14	Stomach	1	358.9° (CW)	180.1°	179°	45°

CW=clockwise; CCW=counterclockwise.

MapCHECK 2 was originally designed to deliver beams en face to the detector surface. In order to simulate a condition where MapCHECK 2 is not affected by the angular dependence, an isocentric mounting fixture (IMF) from SNC was attached to the gantry with MapCHECK 2 and 3 cm solid water block (5 cm water‐equivalent buildup above the detector plane), as shown in Fig. 1(b). The RapidArc QA was performed according to Table 1. Another verification plan for these IMF measurements was conducted by setting the gantry and collimator angles to zero degree in the Eclipse TPS (referred as “IMF QA”).

Planar dose maps on the detector plane for both the MapPHAN QA and IMF QA plans were exported to the SNC patient software (version 6.0) for comparison. The γ passing rate proposed by Low et al.^24^ for each verification plan was determined by the following criteria: 1% (dose difference)/1 mm (distance to agreement (DTA)), 2%/2mm, and 3%/3mm. Absolute dose comparisons with 10% threshold were used for the QA analyses. A two‐tailed Student's t‐test was implemented for statistical analysis at 95% confidence level.

### C. Migration of MapCHECK 2 with IMF

**Figure 2:**
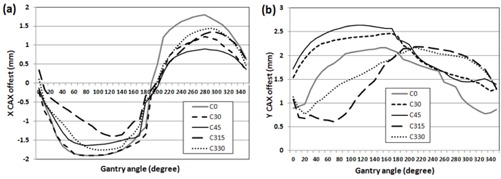
Displacement of central axis (CAX) of MapCHECK 2 on IMF: (a) X offset (MapCHECK 2 lateral) and (b) Y offset (MapCHECK 2 longitudinal) for different collimator angles.

During the QA delivery the central axis (CAX; center diode) of the MapCHECK 2 migrated because of gravity sag of IMF by 7.1 kg of MapCHECK 2 and 2.7 kg of solid water, depending on the gantry angle. The CAX drift was investigated by $10\times 10\;{\text{cm}}^{2}$ MapCHECK 2 measurements on IMF with the 3 cm solid water block for every 10°. The CAX offset was determined by detecting 50% dose levels of normalized X (MapCHECK 2 lateral) and Y (MapCHECK 2 longitudinal) profiles using bilinear interpolation in an in‐house MATLAB program. The CAX offset also relies on the collimator angle and the beam energy. A preliminary study showed that the dependence of CAX offset on energy is inconsequential (less than 0.2 mm for all energies), and thus the offset was investigated with the five different collimator angles using the 6 MV beam and a mean of two measurements was computed. Figure 2 shows the detected CAX displacements of MapCHECK 2 in the X and Y directions. In order to simulate the displacement of CAX, arcs in the IMF QA plans were split every 10° in the Eclipse TPS (only available for version 10 or higher), and the isocenter of each split beam was moved by the CAX offset of a center angle of each angular width (e.g., the CAX offset of 105° for gantry rotation from 100° to 110°). An integrated dose map was exported to the SNC patient software for comparison with the corresponding MapCHECK 2 measurement on IMF (referred as “${\text{IMF}}_{\text{ACTUAL}}$ QA”).

### C. Effect of calibration and effective field size

In order to investigate the effect of calibration field size, the MapCHECK 2 detector array was also calibrated with a field size of $3\times 3\;{\text{cm}}^{2}$ using the four different beam energies. The new calibrations were applied to all the measurements using $10\times 10\;{\text{cm}}^{2}$ calibration for comparison on the SNC Patient software. Clinical acceptance of the measurements using the small‐field calibration was also analyzed with the γ test for the three different γ criteria. However, the γ test based partly on the DTA concept is not a proper comparison metric to fully explore the measurement uncertainty caused by the different calibration. Because the γ test searches a surrounding area of a compared dose point, some points may fail the QA using $10\times 10\;{\text{cm}}^{2}$ calibration but pass the QA using $3\times 3\;{\text{cm}}^{2}$ calibration, even if the dose difference is higher with $3\times 3\;{\text{cm}}^{2}$ calibration. Therefore, the mean normalized dose difference ((Measurement−Calculation)/(Maximum Calculation)×100%)) of all the compared dose points was calculated for each verification plan.

For an in‐depth analysis, effective field sizes of the treatment plans should be presented. An equivalent‐square field size (EqSq) of each control point of the RapidArc plans was determined by a method provided by Nicolini et al.^(25)^ For IMRT fields in the Varian system, the EqSq is calculated by 2×x×y/(x+y) using the mean of all apertures defined by opposing leaves (x) and total leaf width corresponding to the open leaves defined by the jaw settings (y). The mean EqSq from all of the 177 elementary control points per arc for each plan was computed using leaf positions stored in an MLC steering file in DICOM format.

## III. RESULTS

### A. Angular dependence of MapCHECK 2

**Figure 3:**
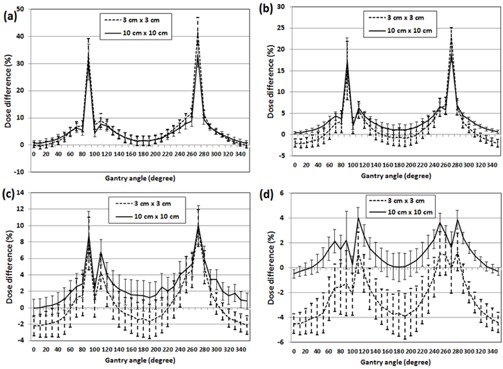
Percent dose difference ((Measurement−Plan)/Plan×100(%)) of MapCHECK 2 measurements with MapPHAN‐MC2. The measurements were performed for every 10° with two different field sizes ($3\times 3\;{\text{cm}}^{2}$ and $10\times 10\;{\text{cm}}^{2}$) and four different energies: (a) 6 MV, (b) 8 MV, (c) 10 MV, and (d) 15 MV. Error bars represent 1 SD of compared points around isocenter (5 points for $3\times 3\;{\text{cm}}^{2}$ and 13 points for $10\times 10\;{\text{cm}}^{2}$).

The angular dependence was considerably high at gantry angles of $90^{\circ }\pm 10^{\circ }$ and $270^{\circ }\pm 10^{\circ }$, as shown in Fig. 3. It decreased substantially from low energy to high energy. For the $10\times 10\;{\text{cm}}^{2}$ field at 90°, the mean differences and 1 SD (13 diode points within the central $2\times 2\;{\text{cm}}^{2}$ field) were 30.6%±6.6% (6 MV), 17.3%±5.3% (8 MV), 8.9%±2.9% (10 MV), and 2.2%±2.3% (15 MV), respectively. For the $3\times 3\;{\text{cm}}^{2}$ field at 90°, the mean differences and 1 SD (five diode points within the central $1\times 1\;{\text{cm}}^{2}$ field) were 33.4%±5.8% (6 MV), 15.0%±6.8% (8 MV), 7.8%±3.2% (10 MV), and −1.3%±2.6% (15 MV), respectively. As reported by Olch,^(21)^ the standard $10\times 10\;{\text{cm}}^{2}$ field calibration underestimated dose for $3\times 3\;{\text{cm}}^{2}$ field measurements compared to $10\times 10\;{\text{cm}}^{2}$ field measurements by 0.6% (6 MV) to 4.0% (15 MV) when the beams were delivered orthogonal to the detector surface (gantry angle of 0°). In general, the difference between $10\times 10\;{\text{cm}}^{2}$ and $3\times 3\;{\text{cm}}^{2}$ field measurements compared to the corresponding TPS calculations was significantly larger for higher energy for most of gantry angles. For 6 and 8 MV, the measurements overestimated dose for the $3\times 3\;{\text{cm}}^{2}$ field even greater than $10\times 10\;{\text{cm}}^{2}$ field at gantry angles of 90° and 270°, whereas for 10 and 15 MV the dose difference for $10\times 10\;{\text{cm}}^{2}$ field was always higher than $3\times 3\;{\text{cm}}^{2}$ field throughout all the gantry angles.

### B. Effect of the angular dependence on the pretreatment QAs

The mean γ passing rates of 14 verification plans were determined for all three γ criteria, as shown in Table 2. For the 3%/3mm criteria, which are most clinically used,^26^, ^27^ 94.0% of QA plans (158 out of 168 compared plans) had the γ passing rate of >95% and all the passing rates were over 90%, except for two outliers (74.5% (Patient #3) and 84.5% (Patient #14) for the 6 MV MapPHAN QA). These two plans had relatively larger field size (mean EqSq: 14.0 cm (Patient #3) and 10.1 cm (Patient #14) for 6 MV). The corresponding γ passing rates of 6 MV ${\text{IMF}}_{\text{ACTUAL}}$ QA were 95.1% (Patient #3) and 99.8% (Patient #14), respectively.

**Table 2:** Mean passing rates (%) of the γ test for the RapidArc QA techniques with $10\times 10\;{\text{cm}}^{2}$ field MapCHECK 2 calibration

		*MapPHAN QA*			*IMF QA*			*IMF_ACTUAL_ QA*		
		1%/1	2%/2mm	3%/3mm	1%/1	2%/2mm	3%/3mm	1%/1	2%/2mm	3%/3mm
6 MV	Mean	55.8	84.6	94.4	60.2	91.0	97.8	57.1	90.4	98.0
	S.D.	18.1	14.9	7.5	8.1	5.4	2.4	8.7	6.1	2.5
8 MV	Mean	77.2	96.7	99.3	56.9	89.8	97.7	57.4	90.8	97.9
	S.D.	12.5	3.6	1.1	7.0	5.4	2.4	7.9	5.6	2.0
10 MV	Mean	74.8	95.0	98.8	62.2	92.6	98.2	59.2	91.8	98.4
	S.D.	14.2	7.4	2.4	7.8	4.1	1.9	9.4	5.8	2.0
15 MV	Mean	81.4	97.9	99.6	58.7	90.1	98.0	58.0	91.3	98.1
	S.D.	11.2	1.7	0.5	7.9	5.4	2.1	8.1	4.8	2.1

S.D.=standard deviation.

For 6 MV, the mean γ passing rate of MapPHAN QA was lower than that of ${\text{IMF}}_{\text{ACTUAL}}$ QA; however, the difference was statistically insignificant (p=0.78(1%/1mm),0.16(2%/2mm), and 0.10(3%/3mm)). For 8, 10, and 15 MV beams, the mean γ passing rates of MapPHAN QA were higher than ${\text{IMF}}_{\text{ACTUAL}}$ QA in all γ criteria. The difference was statistically significant (p<0.05), except the 10 MV QA comparison, using the γ criteria of 2%/2mm(p=0.16) and 3%/3mm(p=0.50). For all the high energies, the difference in the mean γ passing rate between MapPHAN QA and ${\text{IMF}}_{\text{ACTUAL}}$ QA was less than 1.5% for the 3%/3mm criteria. It was amplified as the criteria became stricter (e.g., 81.4%±11.2% (MapPHAN QA) vs. 58.0%±8.1% (${\text{IMF}}_{\text{ACTUAL}}$ QA) using 1%/1 for 15 MV)). The difference in the γ passing rate between IMF QA and ${\text{IMF}}_{\text{ACTUAL}}$ QA was statistically insignificant for all the energies and all the comparison criteria (p‐values from 0.06 (1%/1 for 10 MV) to 0.79 (3%/3mm for 15 MV)), although slightly improved passing rates (∼0.2%) in ${\text{IMF}}_{\text{ACTUAL}}$ QA were observed for the 3%/3mm criteria.

### C. Effect of calibration and effective field size

The mean γ passing rates for different MapCHECK 2 calibration using small field size of $3\times 3\;{\text{cm}}^{2}$ are presented in Table 3. For the MapPHAN QA, they were significantly different from those of $10\times 10\;{\text{cm}}^{2}$ calibration for all the energies, as shown by p‐values in Table 4. For 6 MV. they were lower for the $10\times 10\;{\text{cm}}^{2}$ calibration, while they were significantly higher for 8, 10, and 15 MV. For the IMF QA and ${\text{IMF}}_{\text{ACTUAL}}$ QA techniques, the passing rates of $3\times 3\;{\text{cm}}^{2}$ calibration were not significantly different from those of $10\times 10\;{\text{cm}}^{2}$ calibration for 6, 8, and 10 MV, but they were significantly lower for 15 MV

**Table 3:** Mean passing rates (%) of the γ test for the RapidArc QA techniques with 3 × 3 cm^2^ field MapCHECK 2 calibration

		*MapPHAN QA*			*IMF QA*			*IMF_ACTUAL_QA*		
		1%/1	2%/2mm	3%/3mm	1%/1	2%/2mm	3%/3mm	1%/1	2%/2mm	3%/3mm
6 MV	Mean	57.7	86.1	95.1	59.8	90.6	97.7	56.4	90.3	98.2
	S.D.	18.2	14.0	6.8	8.0	5.4	2.6	8.7	6.2	2.5
8 MV	Mean	61.5	88.9	96.8	60.4	91.6	97.8	60.8	91.7	98.2
	S.D.	18.1	10.7	4.1	10.8	5.3	2.2	9.4	4.8	1.8
10 MV	Mean	62.0	87.1	95.3	61.2	91.9	97.8	58.1	90.7	97.6
	S.D.	20.2	14.7	7.6	9.9	7.0	2.8	8.8	6.8	2.4
15 MV	Mean	54.6	81.1	92.9	50.2	84.2	95.8	47.0	83.5	96.2
	S.D.	18.3	14.0	6.4	8.7	7.6	3.2	6.6	6.1	2.7

S.D.=standard deviation.

**Table 4:** The p‐values of RapidArc QA between $3\times 3\;{\text{cm}}^{2}$ calibration and $10\times 10\;{\text{cm}}^{2}$ calibration of MapCHECK 2

	*MapPHAN QA*			*IMF QA*			*IMF_ACTUAL_ QA*		
	1%/1	2%/2mm	3%/3mm	1%/1	2%/2mm	3%/3mm	1%/1	2%/2mm	3%/3mm
6 MV	<0.01	<0.01	0.01	0.18	0.01	0.94	0.04	0.62	0.05
8 MV	<0.01	<0.01	0.01	0.07	0.02	0.76	0.04	0.15	0.32
10 MV	<0.01	<0.01	0.03	0.47	0.51	0.34	0.52	0.23	0.06
15 MV	<0.01	<0.01	<0.01	<0.01	<0.01	0.01	<0.01	<0.01	<0.01

**Figure 4:**
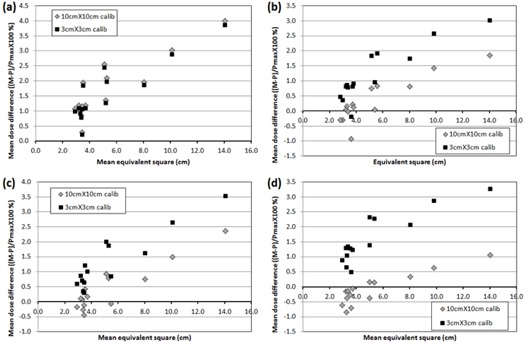
Mean dose error of all compared dose points with respect to mean equivalent‐square field size of RapidArc fields in MapPHAN QA for (a) 6 MV, (b) 8 MV, (c) 10 MV, and (d) 15 MV.

Figure 4 shows the scatter diagram of mean dose error against mean EqSq for MapPHAN QA using the two different calibration field sizes. For 6 MV, the patient‐specific QA measurements were higher than the calculations and the difference between two calibrations was not noticeable. For 8, 10, and 15 MV, the $3\times 3\;{\text{cm}}^{2}$ calibration showed higher dose errors than the $10\times 10\;{\text{cm}}^{2}$ calibration. For plans whose EqSq was around 3 cm, the $10\times 10\;{\text{cm}}^{2}$ calibration generally showed better dose agreement than the $3\times 3\;{\text{cm}}^{2}$ calibration. The $10\times 10\;{\text{cm}}^{2}$ calibration also showed better agreement for plans of larger EqSq (>8cm).

**Figure 5:**
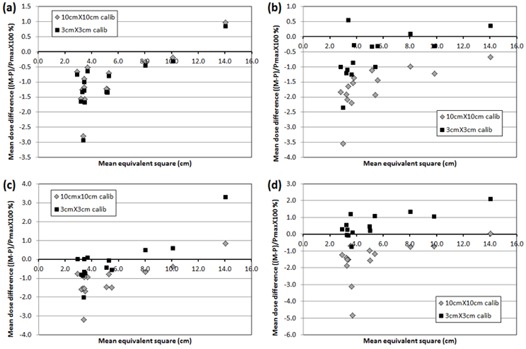
Mean dose error of all compared dose points with respect to mean equivalent‐square field size of RapidArc fields in ${\text{IMF}}_{\text{ACTUAL}}$ QA for (a) 6 MV, (b) 8 MV, (c) 10 MV, and (d) 15 MV.

Figure 5 shows the scatter diagram of mean dose error against mean EqSq for ${\text{IMF}}_{\text{ACTUAL}}$ QA using the two different calibration field sizes. For 6 MV, the absolute difference in the mean dose error between two calibrations was about 0.1% for all the plans. Contrary to MapPHAN QA, for 8, 10, and 15 MV, the plans whose EqSq is around 3 cm showed better agreement using the $3\times 3\;{\text{cm}}^{2}$ calibration. Especially for 10 and 15 MV, the absolute mean dose error was smaller for plans whose EqSq is around 10 cm using the $10\times 10\;{\text{cm}}^{2}$ calibration.

## IV. DISCUSSION

The angular sensitivity change of MapCHECK 2 diode made a noticeable impact on the 6 MV MapPHAN QA. The average γ passing rate of MapPHAN QA was lower than that of ${\text{IMF}}_{\text{ACTUAL}}$ QA (or IMF QA) due partly to two outliers (Patients #3 and #14). The substantially low γ passing rates were observed when the effective field size was relatively large. For the 6 MV beam, the MapCHECK 2 measurement was differentially higher than the Eclipse calculation for all gantry angles (Fig. 3(a)) and it resulted in the higher QA measurements than calculations in the MapPHAN QA for all verification plans, as depicted in Fig. 4(a). The deviation increased as the effective field size became larger. It might possibly be driven by increased scatter components of the large fields.

For higher energies of 8, 10, and 15 MV, all of verification plans were acceptable (γ passing rate of ≥95%) for 3%/3mm criteria using MapPHAN QA and ${\text{IMF}}_{\text{ACTUAL}}$ QA (except for ${\text{IMF}}_{\text{ACTUAL}}$ QA of Patient #6; γ passing rate of ≥91.8%). The difference in mean γ passing rate for 3%/3mm criteria between the two QA methods was not considerably high (Table 2), even if it was statistically significant for 8 and 15 MV. The difference clearly increased as the γ criteria became tighter and IMF QA (or ${\text{IMF}}_{\text{ACTUAL}}$ QA) showed much worse γ passing rates than MapPHAN QA. This can be explained by two sources of uncertainty.

**Figure 6:**
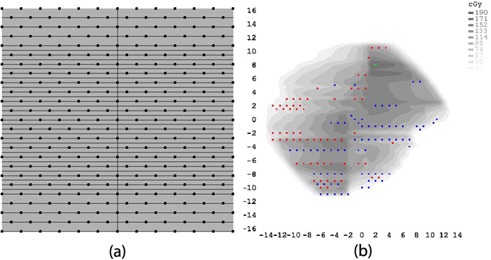
The tongue‐and‐groove effect on the IMF QA demonstrated by continuous hot or cold spots along MLC trajectory: (a) MapCHECK 2 diode points overlaid on the HD‐MLC pattern (inner leaf width =2.5mm, outer leaf width =5mm, and diode size $=0.8\times 0.8\;{\text{mm}}^{2}$), and (b) failed points of 15 MV IMF QA using 2%/2mm criteria for Patient #3. (Red = measurement is high; blue = calculation is high.)

The first source of error is arrangement of MapCHECK 2 diode array. When MapCHECK 2 is used on IMF, rows of diode array are always positioned under gaps between abutting MLC leaves, as shown in Fig. 6(a). This arrangement is strongly susceptible to dosimetric errors caused by the tongue‐and‐groove effect. This effect is clearly demonstrated by continuous hot or cold spots along the rows of MapCHECK 2 diode array, as shown in Fig. 6(b) (15 MV IMF QA using 2%/2mm for Patient #3). Similar problems were observed for Patient #6 whose γ passing rates of IMF QA were considerably lower than other verification plans for high energies. This effect is generally prevalent in RapidArc plans of highly modulated field and large effective field size.

Second, MapCHECK 2 on IMF fundamentally drifts due to gravity sag during gantry rotation. Even if the CAX displacement is simulated with ${\text{IMF}}_{\text{ACTUAL}}$ QA, it cannot be accurately predicted due to discontinuity of every 10° split beams. In addition, the CAX displacement varies even for the same RapidArc deliveries especially with the collimator rotated (e.g., the maximum error of 2.6 mm in the γ direction for collimator $=45^{\circ }$ in Fig. 2(b)). One possible solution for this error source is to deliver all the beams with the gantry arc angle set at 0° used in a research for the Elekta VMAT system, as described in Zhu and Wu.^(23)^ Unfortunately, our current Varian RapidArc system does not allow this delivery method. Khachonkham et al.^(28)^ argued that the complex IMRT plans should be verified by using the IMF QA technique to achieve the QA result with real situation of IMRT treatment rather than single gantry‐angle composite where all beams are delivered with gantry angle of 0°. However, in the study the drift of MapCHECK 2 with IMF was not considered. Our limited study shows that the outcome of IMF QA is not significantly different from that of QA performed in an ideal measurement condition which was, to some degree, simulated by our ${\text{IMF}}_{\text{ACTUAL}}$ QA. Our study also agreed with a conclusion by another study performed by Sweet and Nicolaou^(29)^ where splitting a full arc into partial arcs revealed no unseen failures. However, it is recommended to include the effect of MapCHECK 2 migration on RapidArc QA analysis when IMF is involved, because it depends on the degree of sag (different amount of weight on IMF). Fundamentally, MapCHECK 2 on IMF was intended for conventional IMRT QA. It is not desirable for RapidArc QA without proper consideration of the limitations (gantry rotation information, diode arrangement, and fluence‐based QA), and readers should be cautious about using it for their RapidArc QA.

The angular response of the MapCHECK 2 in this study is similar to other research in which errors were found as large as 25% for 6 MV with the older MapCHECK.^18^, ^20^ The angular response is attributed to design properties of diodes soldered to metal plates on two large circuit boards mounted parallel to one another separated by an air gap and enveloped by two acrylic plates that have conductive surfaces.^(30)^ It can be alleviated by several techniques. Zhang^(22)^ added two types of halfpipe‐shaped boluses (Bolus A: uniform thickness to match the measured dose at the 180° gantry angle, and Bolus B: variable thickness to match the doses at every 15° beam angle) to the planning CT image. The failure rate of γ analysis of six patient QAs was reduced by 39% (Bolus A) and 41% (Bolus B), on average.

The second method is to apply angular correction factors using ratios between detector measurements and corresponding TPS calculated values to modify the measurements.^(23)^ For a typical prostate seven‐fields 6 MV IMRT case, the corrected MapCHECK 2 measurement with the γ criteria of 3%/3mm and 10% threshold gave a 98.7% passing rate compared to 73.1% for the uncorrected measurement. However, it is not practical for RapidArc QA since measurements are not discrete, as in conventional IMRT QA.

Jursinic et al.^(18)^ modified two styles of MapCHECKs by filling air gaps with sheets of Lucite that had custom‐machined slots for the diodes and pieces of copper were added to offset the asymmetric dose sensitivity of diode. They achieved sensitivity change with incident angle less than ±2% using 6 MV. All the studies above were, however, based on one energy (6 MV), and the effect of the calibration field size was not investigated.

Rinaldin et al.^(17)^ showed a good correlation between percentage of lateral fluence and gamma index using the MapPHAN QA, indicating more weighting on the lateral beam results in a worse passing rate (a correlation coefficient r equal to −0.97 for six rectum cases; lateral fluence of 6% to 10% for $90^{\circ }/270^{\circ }\pm 5^{\circ }$). In our study, the average MU ratio (±1SD) for the lateral beams ($80^{\circ }∼100^{\circ }$ and $260^{\circ }∼280^{\circ }$; a mechanical angular ratio $11.1\%(=40^{\circ }/360^{\circ })$) was 13.3%±1.7% (range: 8.7%∼15.9%) using 6 MV, except for Patient #1 (36.9%) for whom two partial arcs were used (the other energies showed the similar ratio). The correlation between lateral fluence and gamma index of the MapPHAN QA was relatively weak for our study (r=−0.16 for 6 MV). This is probably because our QA plans covered a variety of disease sites which yielded different modulation complexity of the RapidArc plans. In this study, even if MapCHECK 2 showed a significant angular dependence, most of QA plans passed the gamma test. In general, it is expected that the γ passing rates are higher with an ideal detector and phantom setup in both MapPHAN and IMF QA. However, unacceptably low γ passing rates were observed in the MapPHAN QA, such as Patients #3 (74.5%) and #14 (84.5%) for the 6 MV MapPHAN QA, even if the plans were clinically acceptable. In this case, additional investigations must be performed using a different QA system. In addition, high γ passing rates do not always mean less clinically relevant patient dose errors. This requires a further study on a correlation between RapidArc QA delivery and clinically relevant patient dose errors which, for example, the Sun Nuclear 3DVH with ArcCHECK QA can provide.

The different field sizes for MapCHECK 2 calibration did not make any remarkable difference for both 6 MV MapPHAN and ${\text{IMF}}_{\text{ACTUAL}}$ QAs, as shown in Figs. 4(a) and 5(a). Even if statistically significant difference was observed in γ passing rate of 6 MV MapPHAN QA for all criteria (Table 4), the absolute difference of the γ passing rate was not substantial (e.g., 94.4%±7.5% ($10\times 10\;{\text{cm}}^{2}$ calibration) vs. 95.1%±6.8% ($3\times 3\;{\text{cm}}^{2}$ calibration) for 3%/3mm). For higher energies (8, 10, and 15 MV), the mean γ passing rate of MapPHAN QA using $10\times 10\;{\text{cm}}^{2}$ calibration was higher than that of $3\times 3\;{\text{cm}}^{2}$ calibration, as seen in Tables 2 and 3. It is attributed to the fact that most of the verification plans (11 out of 14) have the mean effective field size of 5.5 cm or smaller. If the MapCHECK 2 diode array is calibrated with $10\times 10\;{\text{cm}}^{2}$ field but a small field $\left(<5\times 5\;{\text{cm}}^{2}\right)$ is delivered, the measured dose is higher for lateral angles and lower for anterior/posterior angles than the corresponding Eclipse calculations, as shown in Figs. 3(b)‐(d). The error masking reduces the average dose error for small EqSq plans (∼3cm) using the $10\times 10\;{\text{cm}}^{2}$ calibration, as shown in Figs. 4(b)‐(d). However, large EqSq plans (>∼8cm) with the $10\times 10\;{\text{cm}}^{2}$ calibration show overestimation of planned dose because the measurement is higher for most of the gantry angles. The angular dependence of the $3\times 3\;{\text{cm}}^{2}$ measurements using the $3\times 3\;{\text{cm}}^{2}$ calibration is simulated by shifting up the $3\times 3\;{\text{cm}}^{2}$ measurements in Fig. 3 to 0% at gantry angle of 0°. Since the overestimation of small‐field measurement using the $3\times 3\;{\text{cm}}^{2}$ calibration is higher for most of the gantry angles, the dose errors of MapPHAN QA using the $3\times 3\;{\text{cm}}^{2}$ calibration are greater than those of the $10\times 10\;{\text{cm}}^{2}$ calibration. In contrast to MapPHAN QA, the MapCHECK 2 measurement with IMF using the $3\times 3\;{\text{cm}}^{2}$ calibration showed better agreement with the Eclipse calculation for the plans of small mean EqSq (close to 3 cm) for high energies, as shown in Figs. 5(b)‐(d). Interestingly, for 10 and 15 MV, the ${\text{IMF}}_{\text{ACTUAL}}$ QA using $10\times 10\;{\text{cm}}^{2}$ calibration also showed lower dose errors for plans of large mean EqSq of 8 cm or greater (Patients #3, #9, and #14). It is expected that, in the IMF QA, a calibration field size comparable to a mean EqSq of a verification plan will produce a better QA result for high energy (>8MV). It has been demonstrated that the center diode can be calibrated with a reference dose that is delivered with an IMRT calibration plan.^(18)^ Our preliminary study shows that the γ passing rate also depends on the effective field size of the IM calibration field (i.e., the amount of scattered radiations). Since the γ passing rate is not solely dependent upon the calibration method, it will need a further study.

## V. CONCLUSIONS

The angular dependence of 6 MV beam significantly deteriorates the γ passing rate in the MapPHAN QA for some RapidArc plans which produce clinically acceptable passing rate (>95% using 3%/3mm) in the IMF QA. These plans in general have relatively large effective field sizes $\left(>8\times 8\;{\text{cm}}^{2}\right)$. The different calibration field sizes of MapCHECK 2 (conventional $10\times 10\;{\text{cm}}^{2}$ and small $3\times 3\;{\text{cm}}^{2}$) does not have significant impact on both MapPHAN QA and IMF QA using 6 MV. For 8, 10, and 15 MV, the angular dependence does not make any clinically meaningful impact on the MapPHAN QA. However, the $10\times 10\;{\text{cm}}^{2}$ calibration presents slightly better dosimetric agreement for plans of smaller effective field size $\left(<5\times 5\;{\text{cm}}^{2}\right)$ in the MapPHAN QA due to error masking of differential sensitivity for anterior‐posterior beams (underresponse) and lateral beams (overresponse). For the IMF QA, it is recommended that MapCHECK 2 be calibrated using a field size similar to a mean effective field size of a RapidArc plan for better dosimetric agreement. However, it is also expected that the standard $10\times 10\;{\text{cm}}^{2}$ calibration produces clinically acceptable γ passing rate for any field size and energy. Finally, the sagging of MapCHECK 2 on IMF does not make any considerable impact on the γ test for all energies.
